# Decoding cell fate: integrated experimental and computational analysis at the single-cell level

**DOI:** 10.1093/bioinformatics/btaf603

**Published:** 2025-11-05

**Authors:** Yutong Zhou, Shuyang Hou, Xinhao Miao, Guangxin Zhang, Zining Li, Di Zhang, Yongjie Lin, Yihan Lin

**Affiliations:** Integrated Science Program, Yuanpei College, Peking University, Beijing, 100871, China; Integrated Science Program, Yuanpei College, Peking University, Beijing, 100871, China; Integrated Science Program, Yuanpei College, Peking University, Beijing, 100871, China; Integrated Science Program, Yuanpei College, Peking University, Beijing, 100871, China; Integrated Science Program, Yuanpei College, Peking University, Beijing, 100871, China; Peking University Chengdu Academy for Advanced Interdisciplinary Biotechnologies, Chengdu, Sichuan, 610213, China; Center for Quantitative Biology and Peking-Tsinghua Center for Life Sciences, Academy for Advanced Interdisciplinary Studies, Peking University, Beijing, 100871, China; The MOE Key Laboratory of Cell Proliferation and Differentiation, School of Life Sciences, Peking University, Beijing, 100871, China; Peking University Chengdu Academy for Advanced Interdisciplinary Biotechnologies, Chengdu, Sichuan, 610213, China; Center for Quantitative Biology and Peking-Tsinghua Center for Life Sciences, Academy for Advanced Interdisciplinary Studies, Peking University, Beijing, 100871, China; The MOE Key Laboratory of Cell Proliferation and Differentiation, School of Life Sciences, Peking University, Beijing, 100871, China

## Abstract

**Motivation:**

Understanding cell fate determination is crucial in developmental biology and regenerative medicine. Although theoretical frameworks such as epigenetic landscape and gene regulatory networks have been proposed for decades, traditional studies have often been limited by population-averaging and low-throughput techniques, which obscure the heterogeneity of individual cells and fail to provide a systematic view of cell fate control. Recent advances in single-cell technologies have provided unprecedented resolution, revealing the complexity of cell fate decisions and driving the need for more sophisticated computational methods.

**Results:**

In this review, we first emphasize experimental advances, such as single-cell multi-omics, lineage tracing, and perturbation techniques, which produce novel data modalities and enable dynamic tracking of cell fate transitions. We then discuss the modeling paradigms for cell fate studies and further assess the role of emerging AI tools in perturbation modeling and discuss the potential of single-cell and spatial foundation models. Additionally, we highlight several case studies on predicting and manipulating cell fates, and discuss key challenges and future directions of the field.

**Availability and implementation:**

This work generates no new software.

## 1 Introduction

Cell fate determination refers to the process by which individual cells commit to specific functional roles within a multicellular organism, often proceeding through a series of transient or intermediate states (Moris *et al.* 2016, Lee *et al.* 2024). Although this process exhibits intrinsic stochasticity in cellular activities such as gene expression (Losick and Desplan 2008), it nonetheless maintains a high degree of precision. This precision is maintained through the orchestration of tightly regulated signaling pathways and gene regulatory networks, ensuring consistent and evolutionarily conserved outcomes across diverse biological contexts (MacNeil and Walhout 2011).

Significant historical observations have highlighted both the specificity and plasticity of cell fate determination. Spemann and Mangold’s identification of the “organizer” in 1924 marked the first demonstration of inductive signaling in fate determination (Spemann *et al.* 2024). John Gurdon’s demonstration of cell identity plasticity (Gurdon 1962) and Harold Weintraub’s conversion of fibroblasts into myoblasts (Davis *et al.* 1987) further established the feasibility of cell reprogramming. This field reached a watershed moment when Yamanaka and colleagues reprogrammed somatic cells to induced pluripotent stem cells (iPSCs) in 2006 (Takahashi and Yamanaka 2006). Despite these foundational discoveries and subsequent advances in various model systems, the systems-level principles that govern cell fate determination remain incompletely understood (De Belly *et al.* 2022).

To elucidate these mechanisms and predict cell fate determination, substantial theoretical frameworks have been developed. Classical concepts such as Waddington’s epigenetic landscape provide an intuitive metaphor for depicting developmental trajectories, while gene regulatory networks provide a foundation for reaction dynamics-based modeling. Within this framework, numerous models have been proposed, including the mutual antagonistic transcription factor network for myeloid progenitor differentiation (Doré and Crispino 2011) and the seesaw model for stem cell lineage specification (Shu *et al.* 2013). Recent progress has been driven by data-centric techniques enabling the acquisition and analysis of large amounts of single-cell omics data (Tanay and Regev 2017). Advanced sequencing and manipulation techniques now allow detailed profiling of cellular states, capturing both temporal dynamics (Chen *et al.* 2022) and perturbation responses (Peidli *et al.* 2024). Complementing these experimental advances, sophisticated computational algorithms for cell state classification (Pasquini *et al.* 2021), trajectory reconstruction (Kester and Van Oudenaarden 2018), and perturbation-response predictions (Ji *et al.* 2021) have emerged, enabling comprehensive analysis of high-dimensional data and even the generation of new data through generative modeling (Rivero-Garcia *et al.* 2024) for tackling a wide range of questions related to cell fate control.

In this review, we first revisit classical frameworks such as the epigenetic landscape and gene regulatory networks. We then highlight recent progress in single-cell omics and perturbation technologies, followed by computational methods—including AI-driven approaches—for modeling and predicting cell fate. Finally, we discuss emerging applications and future directions bridging data and biology in this rapidly evolving field. By integrating computational and experimental perspectives, this review aims to illuminate the evolving paradigm of cell fate studies and highlight its potential for advancing both basic research and medical applications.

## 2 Theoretical depiction of cell fates

### 2.1 Epigenetic landscape

**Figure 1.**
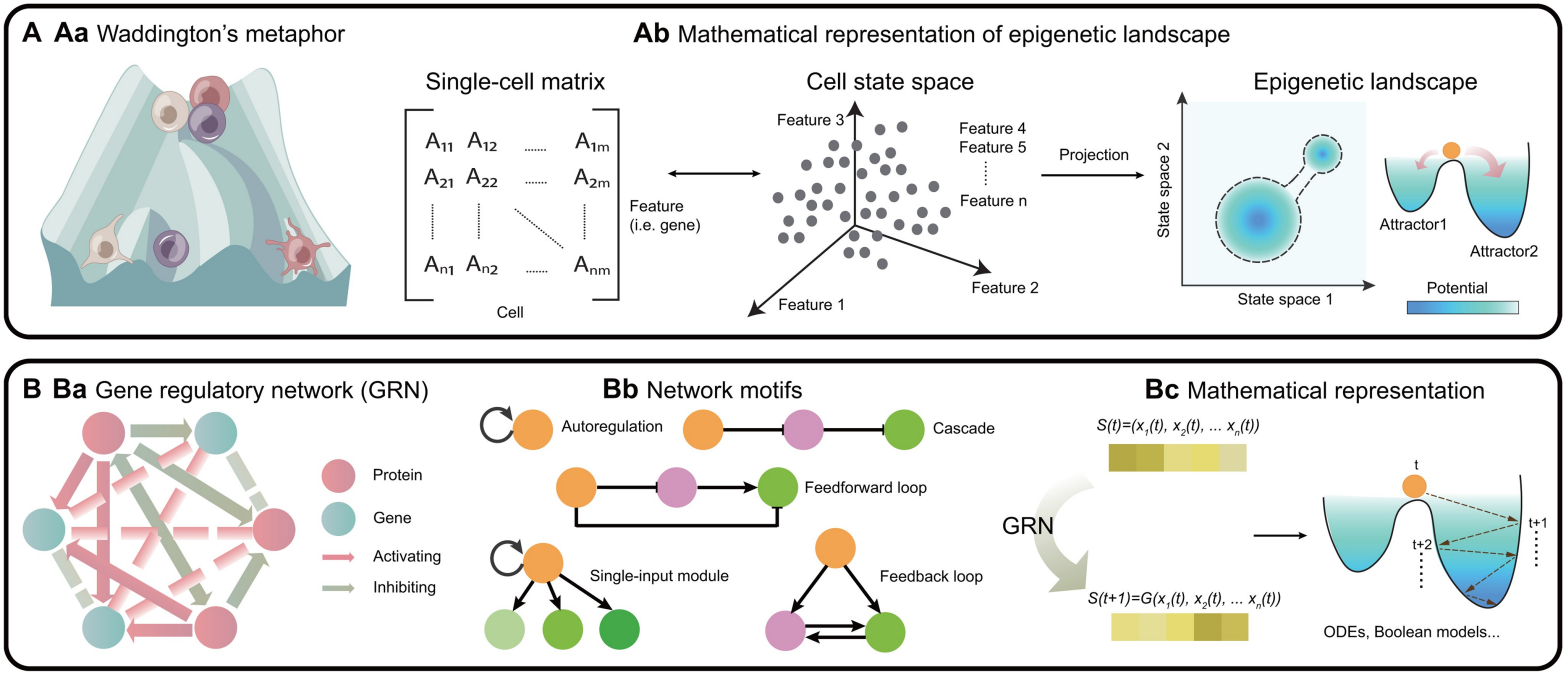
Theoretical depiction of cell fates. (A) Epigenetic landscape. Aa: Waddington’s metaphor. Cells are like stones rolling down a branching hillside, with “valleys” corresponding to distinct cell states. Ab: Mathematical representation of epigenetic landscape. Cell states can be characterized by a single-cell matrix, which turns the epigenetic landscape into a multidimensional phase space. In a three-dimensional projection, each cell fate corresponds to an attractor with a potential denoting its transition probability. Fate transitions can then be conceptualized as cells moving between attractors. (B) Gene regulatory network (GRN). Ba: A schematic of GRN. A GRN depicts genes and proteins as nodes, with edges indicating their regulatory relationships. Bb: Network motifs. Transcription networks are organized through various network motifs of transcription factors and genes, such as autoregulation, cascade, feedforward loops, feedback loops, and single-input modules (Shoval and Alon 2010). Bc: Mathematical representation of GRN. At time t, a cell’s state can be described as a vector $S(t)=(x_{1}(t),\;x_{2}(t),\;\ldots ,\;x_{n}(t))$. The GRN, represented by G, determines how that state evolves over time: $S(t+1)=G(x_{1}(t),\;x_{2}(t),\;\ldots ,\;x_{n}(t))$. This network dynamics can be modeled using ODEs or Boolean approaches. ODE, ordinary differential equation.

Over half a century ago, Conrad Waddington introduced the concept of the “epigenetic landscape” (Fig. 1Aa) (Waddington 1942, 2014). Waddington suggested that the landscape’s formation is governed by underlying gene interactions, where shifts in the regulatory network can alter its contours. Since its inception, this theoretical framework has been continuously refined through concepts from dynamic systems (Kauffman 1969, Ferrell 2012), including attractor and bifurcation theory, providing a mathematical foundation that has evolved into a multidimensional phase space characterized by cell state features (Huang 2012) (Fig. 1Ab).

In the modern view of the epigenetic landscape, stable cell fates represent attractors within the cell state space. Fate transitions can be conceptualized either as cells moving between attractors driven by perturbations, or as reshaping of the landscape itself, where perturbations “lift” or “lower” specific attractors, or even create new ones (Enver *et al.* 2009). The latter scenario is particularly relevant in cancer cells, where new attractors and thus increased attractor variability correspond to heightened plasticity and heterogeneity (Feinberg and Levchenko 2023). Perturbations driving transitions can arise from cellular signals or stochastic fluctuations, and the transitional potential of each fate can be quantitatively approximated using steady-state probabilities (Wang *et al.* 2011) or by decomposing the quasi-potential landscape through vector field analysis (Wang *et al.* 2010, Huang 2012).

### 2.2 Gene regulatory network

To mechanistically decode cell fate decisions, gene regulatory networks (GRNs) offer a powerful framework for modeling how genes and molecular regulators interact to orchestrate cell fate. These networks establish and sustain functional tissues by driving sequential and largely irreversible gene expression patterns leading to specific lineage differentiation (Levine and Davidson 2005).

GRNs are composed of interacting genes, proteins, RNAs and metabolites that participate in the regulatory processes of interest. Among the different types of GRNs, transcriptional networks are especially pivotal, as transcription factors (TFs) serve as key regulators of gene expression during cell fate determination (Spitz and Furlong 2012). Transcription networks are hierarchically organized and highly modular (MacNeil and Walhout 2011), with TFs and their target genes interacting through specific network motifs (Basso *et al.* 2005, Harman *et al.* 2021) (Fig. 1B). These motifs enable precise control over gene expression, allowing cells to integrate diverse signals and adaptively prime for fate decisions. A representative example is the GATA6–NANOG network governing fate decisions between epiblast and primitive endoderm during early mouse development (Fujikura *et al.* 2002, Bessonnard *et al.* 2014). Beyond transcriptional control, post-transcriptional regulation forms another crucial layer, encompassing (i) RNA-binding proteins that modulate splicing, polyadenylation, and mRNA localization (Hentze *et al.* 2018), (ii) microRNAs and long non-coding RNAs that fine-tune transcript abundance and translation (Bartel 2004, Statello *et al.* 2021), and (iii) epitranscriptomic modifications (Roundtree *et al.* 2017). For example, N6-methyladenosine (m6A) modification has been shown to control the balance between self-renewal and differentiation in embryonic stem cells (Geula *et al.* 2015) and embryonic neural stem cell (Wang *et al.* 2018b).

Integrating GRNs into the epigenetic landscape framework allows for mathematical representation of network function. A cell’s state at time t can be generally described as a state vector $S(t)=(x_{1}(t),\;x_{2}(t),\;\ldots ,\;x_{n}(t))$, where $x_{i}(t)$ represents the expression level of gene i. The state at the next time step is given by $S(t+1)=G(x_{1}(t),\;x_{2}(t),\;\ldots ,\;x_{n}(t))$, where function G is determined by the GRN (Lee *et al.* 2024). Over time, cellular states traverse the state space and evolve toward stable attractors in the landscape (Fig. 1Bc). For relatively small-scale GRNs, Boolean models or nonlinear ordinary differential equations (ODEs) are commonly used to model the network dynamics in either a discrete or continuous manner (Karlebach and Shamir 2008). When molecular copy numbers are low, master equations under the Markovian assumption can be used to describe the time evolution of the probabilities of $x_{i}$, thereby capturing the stochastic fluctuation at the single-molecular level (Feinberg and Levchenko 2023).

## 3 Single-cell techniques for investigating cell fates

**Figure 2.**
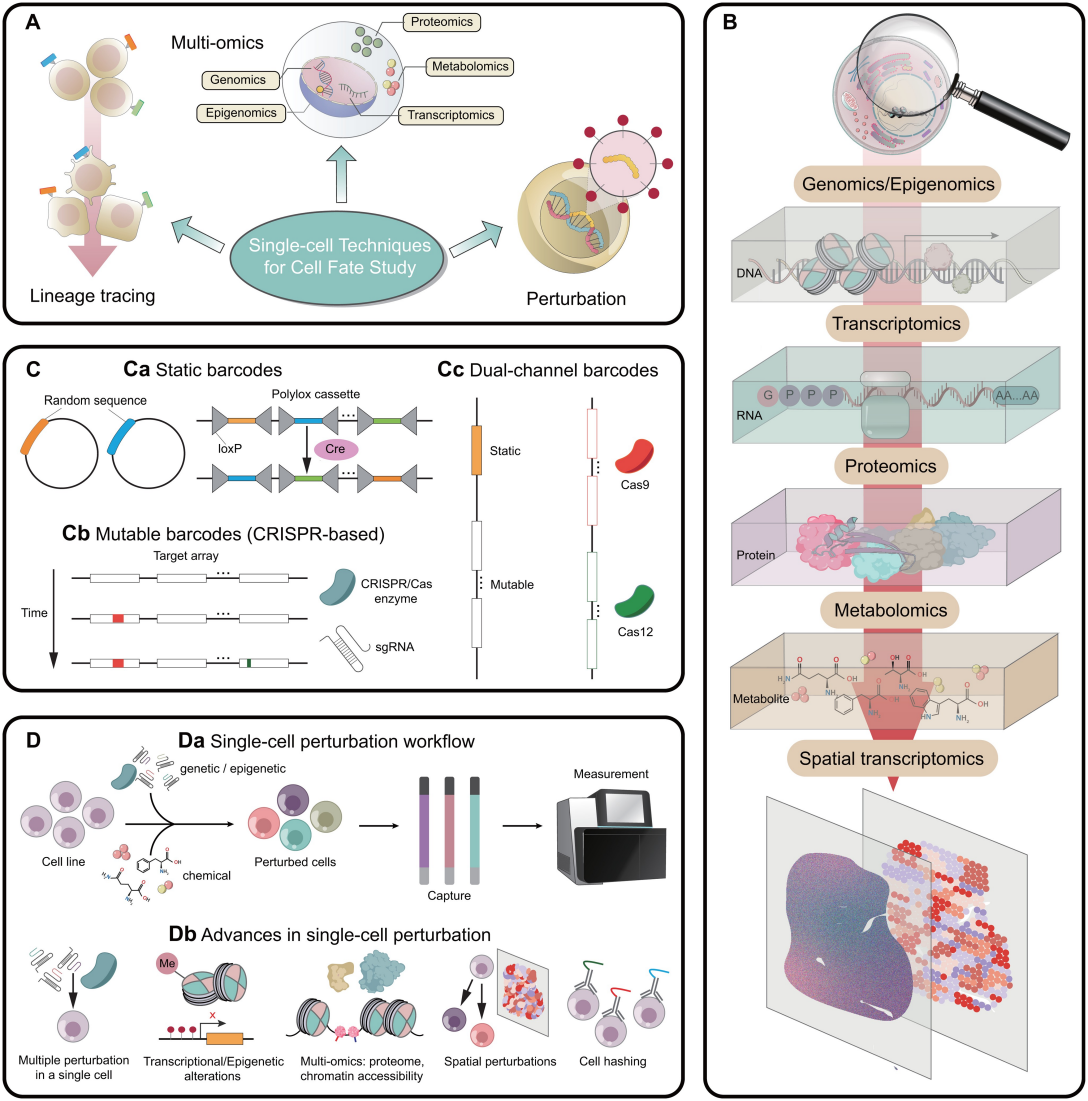
Single-cell techniques for investigating cell fates. (A) Overview of single-cell techniques for cell fate study. Recent advancements of multi-omics, lineage tracing and perturbation at the single-cell level offer a robust experimental framework for investigating cell fates. (B) Single-cell multi-omics. From genomics and epigenomics to metabolomics, these single-cell omics techniques capture cell states at different levels of the central dogma, providing a comprehensive characterization of static cell fates. (C) Various types of barcodes for single-cell lineage tracing. Ca: Static barcodes, including inserted random DNA sequences (Weinreb *et al.* 2020) and polylox barcodes based on the Cre-Lox system (Pei *et al.* 2017). sgRNA, single-guide RNA. Cb: Mutable barcodes, accumulating mutations in target array of the CRISPR-Cas system (Pacesa *et al.* 2024). Cc: Dual-channel barcodes, a combination of static and mutable barcodes systems (He *et al.* 2022, Liao *et al.* 2024) or two orthogonal CRISPR-based barcoding systems (Cas9 and Cas12) (Chen *et al.* 2025a). (D) Single-cell perturbation techniques. Da: Single-cell perturbation workflow. For genetic or epigenetic perturbation, the CRISPR/Cas enzyme and guide RNA (gRNA) pools with guide barcode are delivered into a cell line, and perturbed cells are sequenced to capture gRNAs and assess gene disruption outcomes (Bock *et al.* 2022). For chemical perturbation, small molecules are introduced to single cells, providing experimentally tractable means to shift cell states and probe fate decisions (Gavriilidis *et al.* 2024). Db: Recent advances in single-cell perturbation, including multiple perturbations within single cells (Adamson *et al.* 2016, Dixit *et al.* 2016), integration of transcriptional or epigenetic alterations (Xie *et al.* 2017), multi-omics readouts [e.g. proteome (Mimitou *et al.* 2019, Frangieh *et al.* 2021, Papalexi *et al.* 2021, Wessels *et al.* 2023), chromatin accessibility (Rubin *et al.* 2019, Liscovitch-Brauer *et al.* 2021, Pierce, Granja and Greenleaf 2021)], spatial perturbation, and cell hashing methods (Mimitou *et al.* 2019) to enhance throughput and accuracy.

To accurately capture cell fate attractors and transitions within the epigenetic landscape, experiments should gather extensive phenotypic information as well as dynamic changes at single-cell resolution (Shapiro *et al.* 2013). The emergence of single-cell technologies provide a more quantitative experimental foundation for understanding cell fate decisions, capturing cellular heterogeneity at unprecedented resolution (Fig. 2A) (Han *et al.* 2020).

### 3.1 Multi-modal single-cell omics for comprehensive characterization of static cell fates

The complexity of gene regulatory networks governing cell fate requires genome-level quantification. Next-generation sequencing (NGS) (Margulies *et al.* 2005) has enabled cost-effective, high-throughput analysis of genetic mutations [WGS (Ng and Kirkness 2010)], protein-DNA interactions [ChIP-seq (Park 2009)], transcriptomic patterns [RNA-seq (Wang *et al.* 2009)], and chromatin states [ATAC-seq (Buenrostro *et al.* 2015)] that affect cell fate. Expanding these techniques to the single-cell level has significantly improved the resolution and accuracy of cell fate research. Single-cell RNA sequencing (scRNA-seq) has become a cornerstone of this field, effectively capturing cellular heterogeneity at the transcriptomic level (Kolodziejczyk *et al.* 2015, Tanay and Regev 2017). Besides, single-cell adaptations of classical chromatin-profiling and 3D-genome assays, such as sc-ChIP-seq (Rotem *et al.* 2015), sc-CUT&Tag (Kaya-Okur *et al.* 2019), sc-CUT&RUN (Womersley *et al.* 2025), and sc-Hi-C (Nagano *et al.* 2013), have enabled direct measurement of protein-DNA interactions, histone marks, as wells as higher-order chromatin structure at cellular resolution. Recent technological advancements have further enabled comprehensive single-cell profiling of other biological layers, including chromatin accessibility (Lareau *et al.* 2019), DNA methylation (Luo *et al.* 2017, Chatterton *et al.* 2023), proteome (Specht *et al.* 2021, Ye *et al.* 2025), and metabolome (Yuan *et al.* 2021, Hu *et al.* 2023) (Fig. 2B).

Traditional sequencing-based omics lose spatial context, limiting insights into cell–cell interactions critical for fate decisions (Bressan *et al.* 2023). Emerging spatial omics technologies overcome this by preserving tissue coordinates at single-cell (or near single-cell) resolution (Codeluppi *et al.* 2018, Moffitt *et al.* 2018, Wang *et al.* 2018a), while measuring RNA, proteins, chromatin or multi-modal signals (Takei 2025, Takei *et al.* 2025). Mapping molecular phenotypes back onto three-dimensional tissue architecture, spatial data reveals how physical adjacency (Pelka *et al.* 2021, Kim *et al.* 2023), gradients (Liu *et al.* 2024, Sanchís-Calleja *et al.* 2024), and tissue architecture (Mo *et al.* 2024, Ding *et al.* 2025) bias cell-state transitions. Moreover, integrating spatial omics with lineage tracing (He *et al.* 2022, Xie *et al.* 2023) and perturbation (Dhainaut *et al.* 2022, Baysoy *et al.* 2024, Binan *et al.* 2025) methods further connects where a cell locates and what signals it receives that changes the cell fate, which is critical for understanding biological processes in which cell-cell signaling and position play important roles (Dhainaut *et al.* 2022, Liu *et al.* 2024, Pan *et al.* 2024).

Although integrated single-cell omics provide comprehensive characterization of cell states (Stuart *et al.* 2019, Welch *et al.* 2019), they typically require cell destruction for data extraction, offering only static snapshots (Weinreb *et al.* 2018).

### 3.2 Lineage tracing directly captures cell fate transitions at single-cell resolutions

Reconstructing lineage relationships illuminates authentic cell fate decisions and state transitions, including progenitor-progeny relationships and cellular birth-death dynamics (Quinn *et al.* 2021, Yang *et al.* 2022). Combining lineage tracing with single-cell omics offers a powerful approach to overcome the limitations of static snapshots. These relationships can be traced using pre-genomic methods (e.g. direct observation, tracer dyes, transplantation) or, more recently, genetic barcode-based techniques (VanHorn and Morris 2021).

Two main experimental paradigms exist for establishing lineage relationships in the single-cell era. Retrospective tracing (“labeling-free” methods) leverages naturally accumulated genomic variations, such as single-nucleotide variations (SNVs) (The TRACERx Consortium *et al.* 2017) and copy number variations (CNVs) (Cai *et al.* 2014), in descendant cells to infer lineage relationships based on shared markers. This approach is readily applicable in systems where genetic manipulation is not feasible [e.g. single-cell RNA-seq of patient tumor samples (Wang *et al.* 2014)]. However, the low natural mutation rate limits the number of identifiable genetic features available for lineage tree reconstruction, thus constraining the resolution and accuracy of cell fate studies (Kester and Van Oudenaarden 2018).

In contrast, prospective tracing methods introduce unique genetic barcodes into cells and track their progeny, enabling direct readout of cell lineages. An example is the insertion of random DNA sequences into the genome, which serve as heritable markers detectable alongside scRNA-seq (Fig. 2Ca) (Weinreb *et al.* 2020). The most commonly used systems include Cre-Lox and CRISPR-based barcoding. Cre-Lox lineage tracing is well-suited for in vivo studies, where Cre recombinase acts on polylox cassettes to excise or invert DNA fragments between loxP sites, generating a large repertoire of recombinant barcodes for high-diversity clonal tracing (Pei *et al.* 2017). On the other hand, CRISPR-based barcoding uses Cas9 or other gene editing tools to modify synthetic sequences containing CRISPR target sites (Fig. 2Cb) (Pacesa *et al.* 2024). As lineages expand, these mutable barcodes accumulate unique mutations, providing detailed insights into the hierarchical structure of cellular lineages. However, this can introduce artifacts due to barcode homoplasy and failures in barcode detection, leading to inaccurate lineage tree inferences (Wagner and Klein 2020). On the contrary, static barcodes generated by other techniques provide relatively limited hierarchical information, necessitating additional experiment designs, such as sampling and sequencing cells at multiple time points (Weinreb *et al.* 2020) or implementing multiple rounds of genetic labeling (Biddy *et al.* 2018), often at the expense of data quality. To address these limitations, recent innovations have introduced dual-channel barcoding systems that either integrate static barcoding with mutable CRISPR-based barcoding systems (He *et al.* 2022, Liao *et al.* 2024) or combine two orthogonal CRISPR-based barcoding systems (Chen *et al.* 2025a), offering a more robust experimental foundation for lineage tree reconstructions (Fig. 2Cc).

Single-cell lineage tracing has been widely applied in a wide range of areas, revealing dynamic gene expression properties not captured by static snapshots, such as non-genetic heritable gene expression programs (Schiffman *et al.* 2024), diverse cell state transition trajectories (Harmange *et al.* 2023), and early developmental fate biases (Rodriguez-Fraticelli *et al.* 2018, Wang *et al.* 2022a). Furthermore, lineage tracing is increasingly integrated with single-cell multi-omics in vitro and in vivo (Li *et al.* 2023a, Jindal *et al.* 2024, Liu *et al.* 2025), and also capture cell location and identity with spatial omics (He *et al.* 2022, Koblan *et al.* 2025). However, sequencing-based single-cell lineage tracing is inherently destructive (Tang 2022), precluding repeated measurements from the same cell at different time points or simultaneous profiling of a cell and its progeny. The design of barcodes that can be jointly read out by imaging and sequencing, or the development of live-cell sequencing approaches (Chen *et al.* 2022, Mazelis *et al.* 2025), holds promise for overcoming these limitations.

### 3.3 Perturbation techniques enable cause-and-effect analysis at single-cell resolution

A fundamental challenge in single-cell omics is distinguishing true regulatory causality from correlation in high-dimensional datasets (Crow and Gillis 2018). Directly coupling perturbations with single-cell omics readouts offers a powerful solution (Bock *et al.* 2022), enabling high-dimensional phenotyping and revealing heterogeneous cell responses. Perturbations can be genetic, epigenetic, and chemical (Fig. 2Da).

As with genetic and epigenetic perturbations, linking perturbation identity to single-cell profiles remains critical, typically achieved through paired barcoding (Adamson *et al.* 2016, Dixit *et al.* 2016, Xie *et al.* 2017), gRNA readout constructions (Datlinger *et al.* 2017), or gRNA-targeted capture (Mimitou *et al.* 2019, Replogle *et al.* 2020). Modern perturbation methods extend beyond simple genotype-phenotype mapping (Fig. 2Db). First, Perturb-seq and related methods enable multiple simultaneous perturbations within single cells, increasing screening efficiency and facilitating genetics study (Adamson *et al.* 2016, Dixit *et al.* 2016). Second, CRISPR system can be engineered to induce transcriptional or epigenetic alterations rather than just gene knockouts (Xie *et al.* 2017, Nuñez *et al.* 2021). Third, Combining perturbations with single-cell multi-omics, such as CITE-seq for proteome measurements (Mimitou *et al.* 2019, Frangieh *et al.* 2021) and ATAC-seq for chromatin accessibility (Rubin *et al.* 2019, Liscovitch-Brauer *et al.* 2021), further extends the phenotype space. Moreover, combining perturbations with spatial omics causally maps cellular fate within intact tissue architecture (Dhainaut *et al.* 2022, Binan *et al.* 2025). Lasty, cell hashing can further enhance the throughput and dimensionality of these approaches, enabling simultaneous measurement of even more modalities (Mimitou *et al.* 2019).

Beyond genetic or epigenetic perturbations, chemical perturbations—including small molecules, cytokines or growth factors, and inhibitors or activators—provide experimentally tractable means to shift cell states and probe fate decisions (Gavriilidis *et al.* 2024). Chemical reagents are attractive because they (i) can be delivered transiently or repeatedly without genomic modification, (ii) are often dose-tunable with well-defined concentration–response behavior, and (iii) can be combined or sequenced to produce complex, time-dependent signaling histories that mimic development or therapy. Recent large-scale population compendia [e.g. LINCS/L1000 (Subramanian *et al.* 2017)] and single-cell multiplexing approaches [e.g. sci-Plex (Srivatsan *et al.* 2020), transient barcoding (Shin *et al.* 2019)] offer systematic and high-throughput surveys for multiple compounds, doses, and contexts while controlling for batch effects and reading out heterogeneous responses at single-cell resolution (Cui *et al.* 2024a, McFaline-Figueroa *et al.* 2024). However, it is crucial that the outcome of a chemical perturbation is not just what you add but when, how much, and for how long (Heemskerk *et al.* 2019, Teague *et al.* 2024).

Despite these advances, perturbation workflows face inherent limitations. Determining the precise pre-perturbation state of a cell remains a challenge. Reliable computational inference algorithms (Song *et al.* 2025) or integration with lineage tracing [e.g. in Perturb-seq workflows (Harmange *et al.* 2023)] are needed to fully track perturbation responses from initial to final state. Furthermore, comprehensive genome-wide screens can be limited by sequencing throughput and selecting appropriate targets requires significant biological insight and prior knowledge (Bock *et al.* 2022).

## 4 Modeling paradigms for cell fate determination

In the preceding section, we depicted cell fate within the cell state space in the framework of epigenetic landscape. In the data-rich era, understanding cell fate involves three key steps: defining individual cell states, characterizing transitions between them, and identifying driving factors. We next outline leading approaches for each step across data types.

### 4.1 Constructing a cell state map

**Figure 3.**
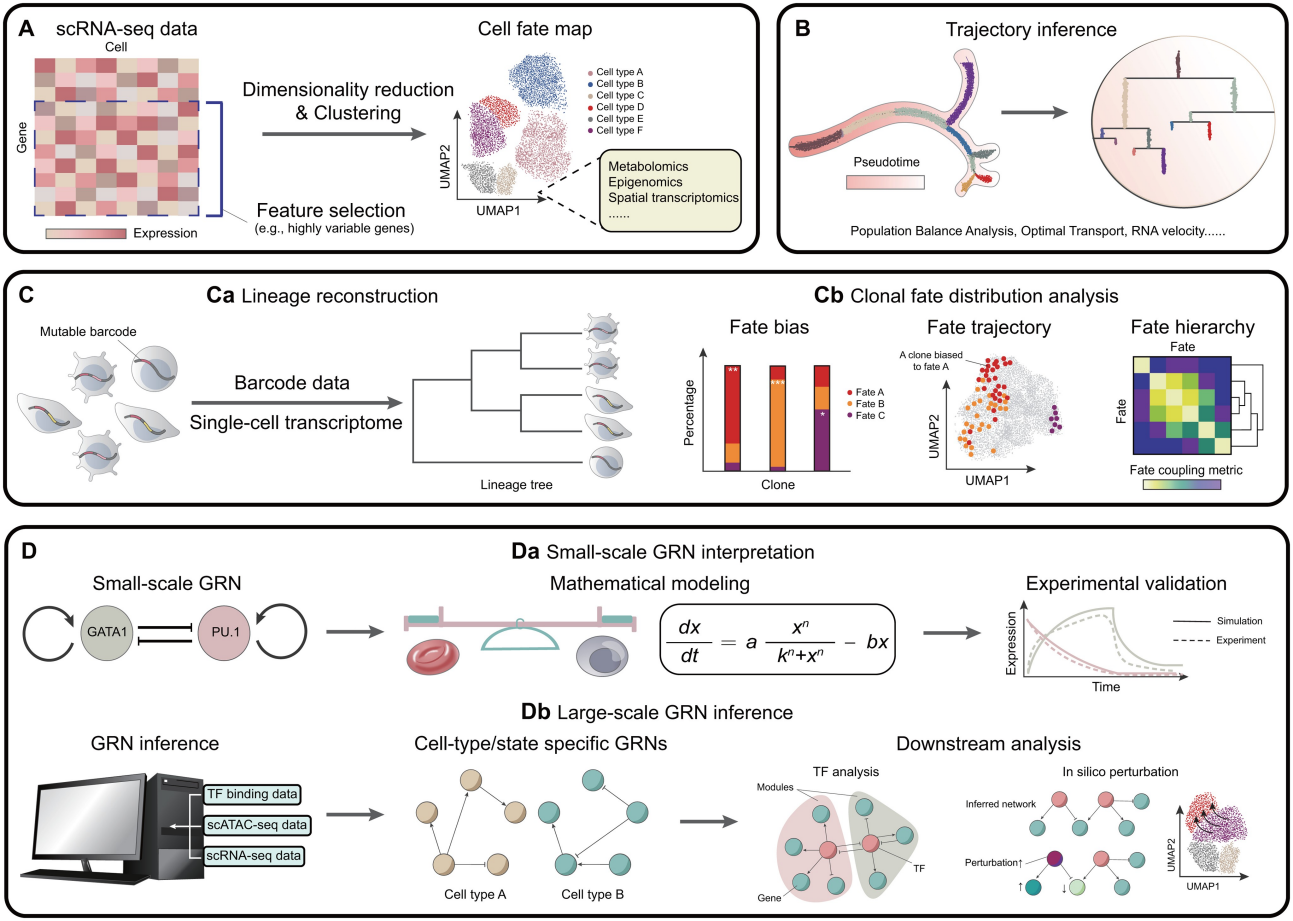
Modeling paradigms for cell fate determination. (A) Constructing a cell fate map. scRNA-seq data are transformed into a cell fate map through feature selection (e.g. highly variable genes), dimensionality reduction and clustering. Each cluster corresponds to an attractor in the gene expression space and is annotated as specific cell type or cell identity based on marker gene expression. Additional omics data, such as metabolomics, epigenomics, and spatial transcriptomics, can also be integrated for deeper functional insights. (B) Trajectory inference from single-cell transcriptomes. Most algorithms infer fate trajectories by ordering cells in pseudotime, a quantitative measure of biological progress (Trapnell *et al.* 2014). Other approaches, including population balance analysis (Tusi *et al.* 2018, Weinreb *et al.* 2018), optimal transport (Schiebinger *et al.* 2019, Sha *et al.* 2024), and RNA velocity (La Manno *et al.* 2018), provide complementary frameworks. (C) Leveraging single-cell lineage tracing. Ca: Lineage reconstruction. Mutable barcode–based lineage tracing requires reconstructing lineage trees from barcode data and/or single-cell transcriptomes. Cb: Clonal fate distribution analysis. Fate bias describes how cells within a particular lineage commit to a dominant fate, with statistical significance assessed against the global distribution (Li *et al.* 2023a, Weng *et al.* 2024). Visualizing clonal cells in a fate map projection (e.g. by UMAP) helps delineate fate boundaries and potentials. Hierarchical clustering based on clonal fate coupling metrics can then unveil fate hierarchies (Rodriguez-Fraticelli *et al.* 2018, Chan *et al.* 2019, Bowling *et al.* 2020, Weinreb and Klein 2020). *, significance marker. (D) Discovering mechanisms through GRNs. Da: Small-scale GRN interpretation. Mathematical models of small regulatory circuits comprising key TFs involved in cell fate determination are derived and experimentally validated. Db: Large-scale GRN inference. Advanced methods integrate TF binding data, scRNA-seq, and chromatin accessibility data (e.g. scATAC-seq) to infer cell-type/state specific GRNs. These inferred networks facilitate downstream analyses, including TF analysis (to identify master regulators via topological metrics) and *in silico* perturbations (to explore fate transitions).

Before assigning a fate for each cell, it is crucial to define the cell state space based on the chosen data modality and the specific biological question. Here, cell states refer to the molecular configurations that cells occupy at a given point in time, while cell fates represent terminal outcomes, the functional roles that cells ultimately adopt. Cell fates are then mapped from cell states based on the premise that cells with similar gene expression profiles share the same state (Rafelski and Theriot 2024). In practice, unsupervised clustering methods, such as Louvain and Ledian algorithm (Traag *et al.* 2019), are used to group heterogeneous single cells around attractors in gene expression space, with each cluster corresponding a distinct cell state (Trapnell 2015). These clusters are subsequently annotated either manually, based on the expression of well-established marker genes, or automatically using annotation tools (Pasquini *et al.* 2021) (Fig. 3A). In the absence of definitive ground truth for cell types, clusters are typically interpreted as distinct cell identities, capturing unique molecular signatures or metabolic states (Wagner *et al.* 2016). It should be noted that some clusters may represent transitioning states, reflecting intermediate or bipotent identity, which should be interpreted with caution.

Despite the widespread use of single-cell transcriptomic data to define cell fates, it is important to recognize that RNA expression alone captures only part of a cell’s phenotypic diversity. Alternative or integrative approaches to mapping cell fates—incorporating metabolomics (Rombouts *et al.* 2021), epigenomics (Liao *et al.* 2024, Zuo *et al.* 2024), or spatial information (Srivatsan *et al.* 2021, Li *et al.* 2024b)—may offer more comprehensive insights. For example, sci-Space applied to developing mouse embryos enabled the reconstruction of spatially resolved cell state trajectories and migratory patterns of differentiating neurons (Srivatsan *et al.* 2021). Moreover, to alleviate the “curse of dimensionality,” it is often necessary to perform feature selection (e.g. highly variable genes) and apply dimensionality reduction methods prior to clustering. However, each of these steps introduces additional parameter choices that bring in variability, potentially causing misclassification of cell states and the inadvertent omission of rare populations (Patterson-Cross *et al.* 2021).

### 4.2 Trajectory inference from single-cell transcriptomes

Once cell fates have been defined, the subsequent step is to characterize the fate trajectory. A major class of methods for this purpose, known as trajectory inference (TI), posits that accurate prediction of fate trajectory is possible if an adequate number of cells in transitional states are captured (Kester and Van Oudenaarden 2018). The first category of methods assumes that cells with similar expression profiles are closely positioned along the continuum of fate transitions, an idea rooted in the concept of pseudotime, which was originally proposed by Monocle (Trapnell *et al.* 2014). Building on this premise, numerous algorithms arrange cells along a pseudotime axis from snapshot data according to various criteria (Kester and Van Oudenaarden 2018) (Fig. 3B). A second category of methods conceptualizes fate transitions as a stochastic process and leverages time-series scRNA-seq to reconstruct trajectories (Weinreb *et al.* 2018, Schiebinger *et al.* 2019, Yeo *et al.* 2021, Sha *et al.* 2024). Notably, Waddington-OT uses optimal transport to infer the temporal evolution of cell state distribution during cellular reprogramming and reveals a broader spectrum of developmental programs (Schiebinger *et al.* 2019). Beyond these approaches, RNA velocity (La Manno *et al.* 2018) offers another way to infer the direction and likelihood of cell state transitions by estimating the time derivative of gene expression—captured through the ratio of spliced to unspliced mRNA. This concept has been extended to account for transient cell states (Bergen *et al.* 2020) and continuous vector fields by incorporating metabolic labeling scRNA-seq (Qiu *et al.* 2022).

Despite the utility of these methods in discerning kinship and transitions among cell types, multiple challenges persist. First, the assumption that cells with similar transcriptomes are temporally proximal may be confounded by factors such as cell cycle dynamics, spatial constraints, and cellular stress (Tritschler *et al.* 2019). Second, many trajectory methods assume fixed starting, branching, and ending points, which may not accurately reflect complex differentiation patterns (Saelens *et al.* 2019). Moreover, branching inferred from transcriptomic data does not necessarily coincide with actual cell division events and need not form a strictly tree-like structure, potentially leading to misinterpretations such as spurious branch points or overlooked dynamic processes (Wagner and Klein 2020).

### 4.3 Leveraging single-cell lineage tracing

Single-cell lineage tracing can address many of these limitations by coupling single-cell omics with genetic labeling, thereby capturing both phenotypic characteristics and lineage information within the same sample. This introduces an additional step of lineage data processing. For static barcode, clones are called based on unique barcode (or combinations); for mutable barcode, specialized bioinformatic tools are developed to infer discrete lineage tree structure (Fig. 3Ca). Some algorithms reconstruct lineage trees solely from the barcode data (Jones *et al.* 2020, Sashittal *et al.* 2023), while others (Zafar *et al.* 2020, Pan *et al.* 2023) integrate transcriptomic information into the reconstruction process. The performance of these methods has been benchmarked (Gong *et al.* 2021).

Because fate determination is inherently dynamic, the characterization of fate trajectory typically focuses on changes in clonal fate distributions as the lineage expands (Fig. 3Cb). Many lineages exhibit fate bias, reflecting their tendency—whether through intrinsic mechanisms or stochastic processes—to produce specific cell fates (Weinreb *et al.* 2020, Weng *et al.* 2024). At the clonal level, fate bias can be quantified by the proportion of cells that adopt the dominant fate, with statistical significance can be evaluated against the global fate distribution (Li *et al.* 2023a, Weng *et al.* 2024). When visualized on a cell state map, these clonal trajectories form distinct paths, further illustrating fate boundaries and potentials. Moreover, relationships among different fates can be inferred by hierarchical clustering based on clonal fate coupling metrics (Rodriguez-Fraticelli *et al.* 2018, Chan *et al.* 2019). On the other hand, when explicit lineage tree is available, permutation tests (shuffling fate labels across the same tree structure) can be used to assess fate bias (Regalado *et al.* 2025), evaluate the heritability and correlation of cells states (Schiffman *et al.* 2024), and identify recurrent lineage motif (Tran *et al.* 2024). Additionally, several studies have established mathematical models to describe the dynamic process of clonal fate transitions using transition maps (Wang *et al.* 2022a) and optimal transport (Forrow and Schiebinger 2021, Lange *et al.* 2024).

It is important to note, however, that cells within the same lineage do not necessarily exhibit similar expression profiles, and cell division and differentiation are not always perfectly coupled (Udomlumleart *et al.* 2021, Kukreja *et al.* 2024). Therefore, caution is warranted when using gene expression data to assist in lineage tree reconstruction or when inferring trajectories based solely on lineage information.

### 4.4 Discovering mechanisms through GRNs

After mapping cell fate trajectories, studies often explore underlying mechanisms via GRNs, using either small-scale modeling of known TFs or large-scale inference. Leveraging previously identified core TFs relevant to specific biological questions, many studies have used mathematical modeling followed by experimental validation to probe small-scale GRNs (Fig. 3Da). Such work has yielded crucial insights into a range of differentiation processes, including the binary fate decision between erythroid and myelomonocytic lineages governed by GATA1 and PU.1 (Huang *et al.* 2007), cellular reprogramming (Chickarmane *et al.* 2006), and cell polarization (Chau *et al.* 2012).

With the accumulation of biological knowledge and advances in high-throughput techniques, reconstructing large-scale GRNs has become a major focus in systems biology. Since the essence of transcriptional network inference lies in defining the relationships between TFs and their target genes, the most straightforward approach is to evaluate correlations from transcriptome data and identify co-expressed gene modules (Langfelder and Horvath 2008). To distinguish true regulatory interactions from spurious correlations and establish regulatory directions, TF binding profiles [i.e. measured by ChIP-seq (Park 2009) and CUT&RUN (Skene and Henikoff 2017)] or chromatin accessibility data are typically used to assign TFs to cis-regulatory elements based on motif analysis. Then, the regulatory elements are linked to target genes within a certain genomic distance (Badia-i-Mompel *et al.* 2023). In the single-cell field, state-of-the-art methods use scRNA-seq and scATAC-seq to reconstruct GRNs for distinct cell states (Fig. 3Db) and they have been comprehensively reviewed and benchmarked elsewhere (Badia-i-Mompel *et al.* 2023, Nourisa *et al.* 2025). Besides these multimodal approaches, some methods infer GRNs using perturbational scRNA-seq data (Ishikawa *et al.* 2023, Jiang *et al.* 2023, Littman *et al.* 2023, Hu *et al.* 2025) for casual regulatory relationships. Once inferred, GRNs can significantly enhance our understanding of cell fate in at least two ways. First, network topology analyses (e.g. TF centrality) can reveal hubs and modules in the network and pinpoint key regulators (Kuppe *et al.* 2022, Lee *et al.* 2024), while enrichment-based strategies infer TF activity from transcriptomics data to uncover the roles of master regulators in fate decisions (Aibar *et al.* 2017, Walsh *et al.* 2017, Garcia-Alonso *et al.* 2018). Second, GRNs can be used to predict fate transitions, exemplified by CellOracle (Kamimoto *et al.* 2023) and SCENIC+ (Bravo González-Blas *et al.* 2023), which performs in silico TF perturbations to estimate cell identity transition probabilities (Qiu *et al.* 2022).

However, several challenges remain in GRN inference. First, TF motif databases are often incomplete, especially for members of the same family and for co-binding events (Inukai, Kock and Bulyk 2017). Moreover, chromatin accessibility does not necessarily imply TF binding; complementary information from scChIP-seq (Rotem *et al.* 2015) and scCUT&Tag (Kaya-Okur *et al.* 2019, Bartosovic *et al.* 2021) can help address this limitation, and chromosome conformation assays such as single-cell Hi-C (Nagano *et al.* 2013) provide additional insights into long-range regulatory connections. Third, regulatory effects can shift due to epigenetic changes like promoter DNA methylation, which has been ignored in current frameworks (Fan *et al.* 2024). In addition, most mainstream GRN inference methods focus primarily on transcriptional networks; integrating post-transcriptional elements such as miRNAs (Kittelmann and McGregor 2019, Li *et al.* 2025) and alternative isoforms (Lambourne *et al.* 2025) could further enhance the resolution of inferred regulatory programs.

## 5 AI-driven advances in understanding cell fates

In recent years, rapid advances in artificial intelligence (AI) have profoundly reshaped scientific research(Embedding AI in Biology 2024). In the realm of cell fate study, AI-driven models have gone beyond conventional applications such as cell type annotation (Pasquini *et al.* 2021) and GRN inference and response predictions (Mochida *et al.* 2018), and are beginning to establish a new paradigm: the construction of AI virtual cells (AIVCs) (Bunne *et al.* 2024, Carr *et al.* 2024, Noutahi *et al.* 2025). By integrating extensive datasets into large-scale machine learning frameworks, AIVCs are envisioned as computational surrogates capable of simulating cellular behavior across multiple scales and diverse states (Bunne *et al.* 2024). Within the context of cell fate, the ability of AIVCs to represent and discover novel cellular states, and to conduct in silico experiments that manipulate these states, is be particularly valuable, as it can narrow down the hypothesis space and accelerate biological discoveries. In this section, we highlight recent advances toward this vision with a focus on perturbation modeling and single-cell foundation models.

### 5.1 Perturbation modeling

Perturbation modeling aims to predict cellular responses and uncover mechanisms based on genetic or chemical inputs and phenotypic readouts (Gavriilidis *et al.* 2024). A wide array of classical machine learning models has been developed to analyze perturbation effects, with targets ranging from quantifying perturbation magnitude (Dixit *et al.* 2016, Yang *et al.* 2020, Dong *et al.* 2023), ranking the perturbation effects (Duan *et al.* 2019, Yang *et al.* 2020), and finding optimal perturbation strategy (Zhang *et al.* 2023) to identifying distinct cell groups or states (Jin *et al.* 2022, Hawkins *et al.* 2023) and prioritizing responsive cell types (Skinnider *et al.* 2021). Compared with purely statistical approaches, these machine learning models are generally more adept at extracting meaningful features from large-scale, high-dimensional datasets, and they still maintain high interpretability due to the simplicity of structure and transparency. Nonetheless, their performance hinges on effective feature engineering, which can be constrained by the need for prior knowledge exceeding our current biological understanding (Li *et al.* 2023b), and they often lack the ability to perform in silico prediction at scale.

Through hierarchically structured networks, deep neural networks (DNNs) surpass the constraints of shallow learning approaches by uncovering previously unknown patterns and constructing rich latent space representations in a data-driven manner (LeCun *et al.* 2015). Perturbation modeling with DNNs is typically framed as learning latent representations in which baseline cell states are transformed by perturbation embeddings to generate predicted phenotypic outcomes. Genetic perturbation are often encoded as gene embeddings, whereas chemical perturbations rely on molecular descriptors with dosage information (Hetzel *et al.* 2022). Despite the differences, most models converge on autoencoder-based architecture (Roḳaḥ *et al.* 2023). By perturbing the latent layer directly (Sadria *et al.* 2024) or comparing the latent space representations of perturbed and unperturbed cells (Keinan *et al.* 2023), these models can evaluate the perturbation effect.

**Figure 4.**
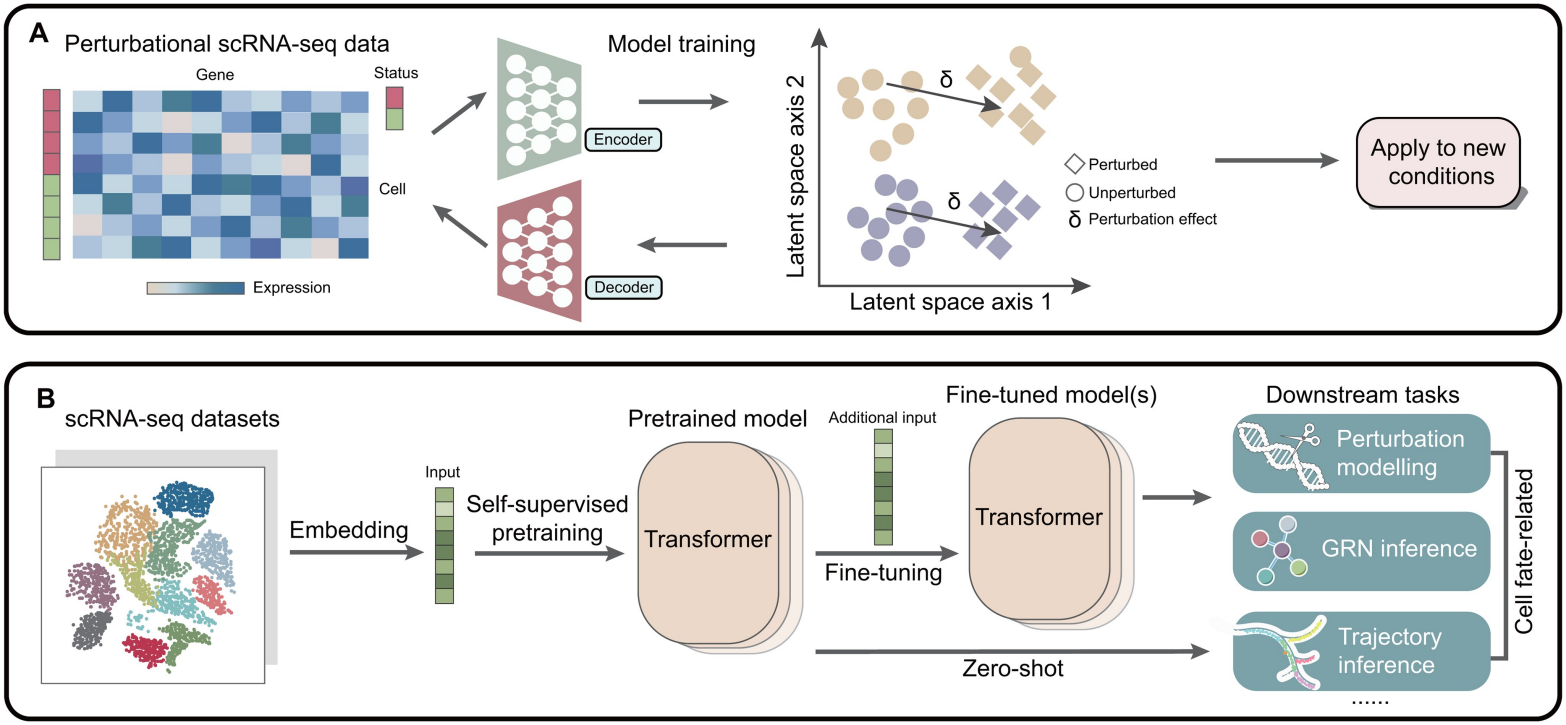
AI-driven advances in understanding cell fates. (A) A schematic representation of scGen model (Lotfollahi *et al.* 2019). scGen utilizes a variational autoencoder trained on scRNA-seq data from both perturbed and unperturbed cells, mapping these cells within a latent space. The shift (δ) between perturbed and unperturbed representations reflects the differences in cellular states due to perturbations, which can be applied to novel conditions (such as new cell types). (B) Overview of a single-cell foundation model. scRNA-seq datasets are embedded as inputs for self-supervised pretraining of a transformer model. After pretraining, the model can perform cell-fate related downstream tasks such as perturbation modeling, GRN inference and trajectory inference, either through fine-tuning or in a zero-shot manner.

Standard autoencoders struggle to generalize to unseen perturbations or cell types, whereas variational autoencoders (e.g. scGen) enable such predictions by learning probabilistic latent spaces that capture transferable perturbation effects (Fig. 4A) (Lotfollahi *et al.* 2019). Subsequent VAE-based methods enhance model capability or interpretability through various strategies, such as refining VAE architectures (Lotfollahi *et al.* 2019, Rampášek *et al.* 2019), evaluating perturbation effects in a cell-type-specific manner (Kana *et al.* 2023, Weinberger *et al.* 2023), and integrating biological insights from gene annotations (Seninge *et al.* 2021), ontology information (Doncevic and Herrmann 2023), or functional enrichment analysis (Geiger-Schuller *et al.* 2023). Meanwhile, the compositional perturbation autoencoder (CPA) (Lotfollahi *et al.* 2023) uses an adversarial autoencoder framework to decompose the data into a collection of latent embeddings for cell type, perturbations and other covariates, enabling prediction the effects of new perturbation combinations. Advance CPA versions have introduced specialized networks for encoding small molecules (Hetzel *et al.* 2022) or incorporating multi-modal data (Inecik *et al.* 2022). Beyond autoencoders, other types of neural networks have also demonstrated impressive capabilities in perturbation-focused tasks (Bunne *et al.* 2023, Zheng *et al.* 2023, Piran *et al.* 2024, Roohani *et al.* 2024, Zinati *et al.* 2024). A notable example is GEARS (Roohani *et al.* 2024), which predicts perturbation responses by learning gene perturbation embeddings derived from a gene ontology knowledge graph combined with an inferred gene co-expression network. More recent work further leverage these strategies via subtask-decomposition (Gao *et al.* 2024) or introducing attention mechanisms (Alsulami *et al.* 2024, Bai *et al.* 2024).

Despite their promise, deep learning models for perturbation prediction face notable limitations. Benchmarking studies show that they often fail to outperform linear baselines (Wu *et al.* 2024, Ahlmann-Eltze *et al.* 2025), and may approach random guessing in zero-shot predictions (Li *et al.* 2024a). A plausible explanation is that many perturbations impact only a small subset of genes and have limited downstream effects on the broader gene network, making simple additive linear models sufficiently effective for capturing global expression profiles (Rood *et al.* 2024). The recent Virtual Cell Challenge represents an important step toward community-wide benchmarking for better model development (Roohani *et al.* 2025). As data resources and evaluation frameworks continue to expand, collective efforts are expected to steadily improve model robustness and accelerate progress toward realistic digital virtual cells.

### 5.2 Foundation models

Due to the inherent similarities between natural language and single-cell omics data, particularly in their sequential structure and context dependency, large language models (LLMs) have recently been applied to single-cell data analysis. These models, also referred to as single-cell foundation models (scFMs), are typically built upon the self-attention transformer architecture and pre-trained on extensive single-cell RNA-seq datasets (Szałata *et al.* 2024) (Fig. 4B). Although these models were not originally designed for cell fate research, many of their downstream applications, such as perturbation modeling and GRN inference, lend themselves naturally to investigating cell fate. More recently, similar foundation model frameworks have been extended to spatial transcriptomics, which will also be discussed. For further details on the architectures, pre-training strategies, or applications in other areas, we refer readers to recent reviews (Consens *et al.* 2023, Szałata *et al.* 2024).

Compared with the deep neural networks introduced earlier, scFMs bring two key advantages to perturbation modeling: comprehensive cell-state embeddings learned from extensive datasets, and the ability to capture complex gene-gene interactions through attention mechanisms. Typified by scFoundation (Hao *et al.* 2024a), some models (Gong *et al.* 2023, Liu *et al.* 2023a, Yang *et al.* 2024, Hao *et al.* 2024a) adopt a straightforward approach by directly inputting embeddings into other advanced perturbation prediction models like GEARS. In this setup, GEARS treats these embeddings as nodes in a gene-relational graph and integrates them with perturbation information to predict post-perturbation gene expression. Other models (Amara-Belgadi *et al.* 2023, Wen *et al.* 2023, Cui *et al.* 2024b, Hsieh *et al.* 2024) opt to fine-tune with Perturb-seq data, adapting their representations to enhance predictive capabilities. An alternative is the zero-shot approach showcased by Geneformer (Theodoris *et al.* 2023). Here, genes are ranked by normalized expression, and perturbation is simulated by moving a gene to the top of the ranking (activation) or removing it (inhibition). By measuring the cosine similarity between original and perturbed embeddings for both cells and genes, Geneformer can quantify the potential effect of perturbing each gene in its cellular context.

Beyond perturbation modeling, scFMs are also increasingly applied to GRN inference. Here, the self-attention mechanism provides a natural advantage: each attention head produces weights that quantify how strongly one gene’s representation attends to others, effectively capturing putative regulatory dependencies. By aggregating these weights across layers or heads, one can construct a gene–gene similarity network that reflects context-specific transcriptional regulation. Typical workflows involve (i) extracting the attention matrix of genes, (ii) constructing a similarity network based on attention patterns similarity, and (iii) clustering genes to identify commonalities in function or expression (Shen *et al.* 2022, Amara-Belgadi *et al.* 2023, Cui *et al.* 2024b, Kalfon *et al.* 2025). Additionally, scGPT (Cui *et al.* 2024b) offers a complementary approach by comparing a gene’s attention scores before and after perturbation using Perturb-seq data, enabling the construction of subnetworks centered on perturbed genes and highlighting casual regulatory effects.

Building on these advances, foundation models have also been extended to spatial transcriptomics with the rapid development of the technology. Compared with scFMs, spatial foundation models (spFMs) aim not only to represent gene expression states but also to capture their spatial organization within tissues. spFMs typically adopt graph-based frameworks [e.g. NicheCompass (Birk *et al.* 2024) and Novae (Blampey *et al.* 2024)] or transformer-based architectures [e.g. Nicheformer (Schaar *et al.* 2024), stFormer (Cao *et al.* 2024), scGPT-spatial (Wang *et al.* 2025) and SToFM (Zhao *et al.* 2025)] and are pretrain on large spatial transcriptomics datasets. Through this process, they learn joint embeddings that integrate transcriptomic similarity with spatial proximity, thereby enabling a range of downstream tasks such as cell type prediction and deconvolution (Schaar *et al.* 2024, Wang *et al.* 2025, Zhao *et al.* 2025), gene expression imputation (Wang *et al.* 2025, Zhao *et al.* 2025), and signaling pathway analysis (Birk *et al.* 2024, Blampey *et al.* 2024). Besides, some models introduce multimodal extensions by combining spatial transcriptomics with histology images (Chen *et al.* 2025b, Liu *et al.*, Zhang *et al.*) or proteomics (Madhu *et al.* 2025), thereby enriching the learned embeddings with complementary structural and molecular information. Collectively, these efforts highlight the potential of spFMs to serve as general-purpose models for tissue-scale biology, bridging molecular states with spatial context.

Although foundation models do not fundamentally change the underlying principles of cell fate research, they offer new methodologies that leverage larger datasets and advanced model architectures to create more expressive data representations. However, empirical evidence suggests that task-specific models can often perform on par with or even exceed the performance of transformer-based approaches (Kedzierska *et al.* 2025). For example, scFMs still lag behind simpler methods like GEARS in genetic perturbation tasks, and do not consistently excel in GRN inference (Liu *et al.* 2023b). They also face challenges such as data scarcity, the nonsequential nature of single-cell omics data, sensitivity to hyperparameters, interpretability and high computational cost (Consens *et al.* 2023). However, with the emergence of spatial foundation models, we anticipate a new wave of models that integrate multimodal, multi-omics, and spatiotemporal data, moving toward uniform representations of cells that could serve as the basis for an AIVC.

## 6 Case studies of applications

**Figure 5.**
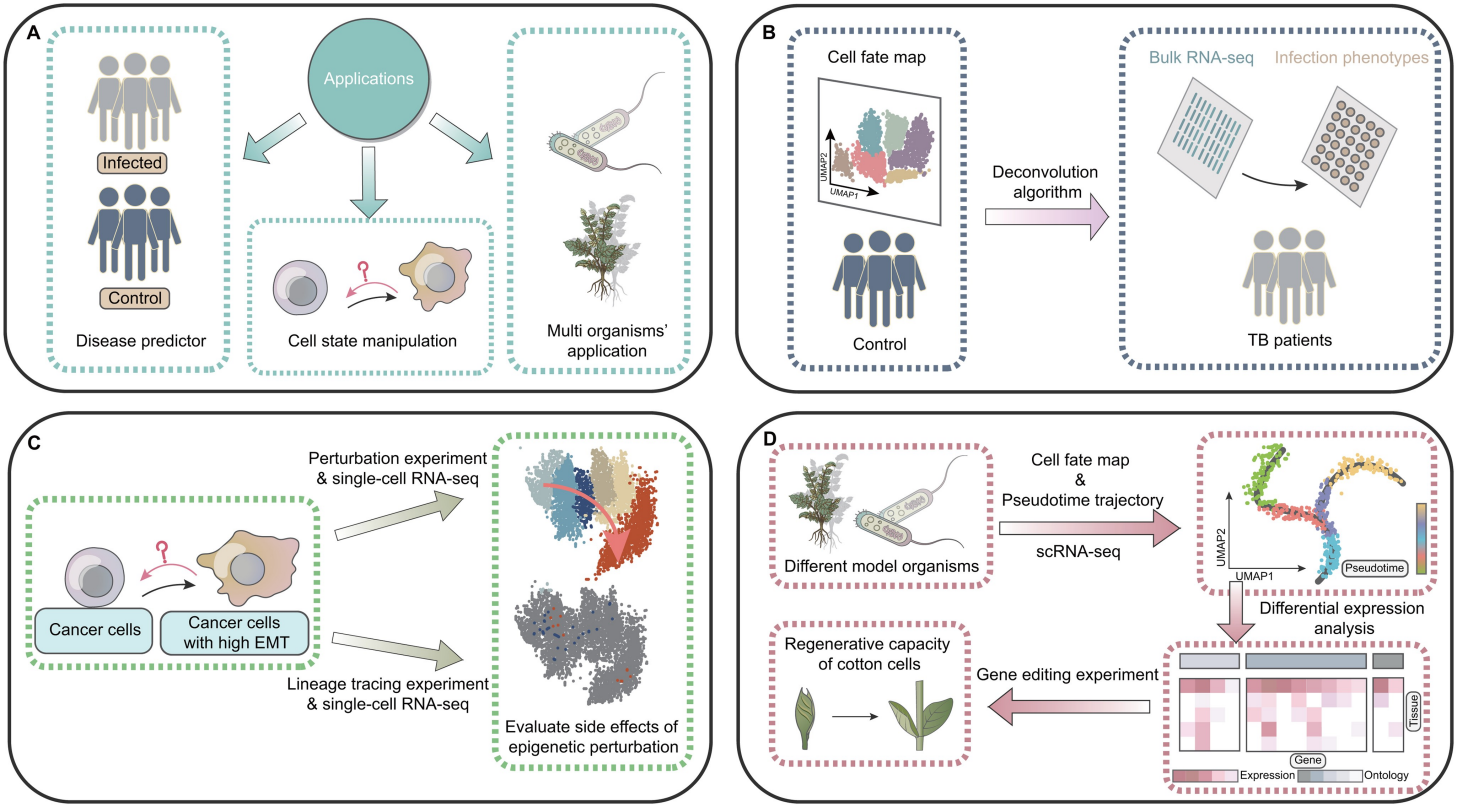
Case studies of applications. (A) Overview of application domains in cell fate research. (B) Deconvolution of bulk RNA-seq data using scRNA-seq profiles from healthy and infected samples allows the inference of disease-induced, cell-type-specific immune responses of tuberculosis (TB) (Bossel Ben-Moshe *et al.* 2019). (C) Cell state manipulation for cancer therapy. In cancer models, perturbation and lineage tracing experiments, combined with single-cell RNA-seq, reveal the side effects of epigenetic manipulations (Schaff *et al.* 2024, Metzner *et al.* 2025). Such studies help identify potential therapeutic targets and can also inform regenerative medicine research. (D) Expansion of single-cell techniques to multiple model organisms. From investigating plant regenerative capacity (e.g. in cotton) to other species, scRNA-seq and pseudotime analysis generate cell fate maps for comparing developmental processes (Zhu *et al.* 2023). Follow-up gene editing experiments, guided by differential expression and ontology analyses, serve to validate predicted regulatory factors and highlight evolutionarily conserved mechanisms.

As previously discussed, single-cell technologies and their associated analytical methods have been widely adopted in laboratories, greatly advancing our understanding of the mechanisms underlying cell fate determination. In this section, we will explore several case studies that illustrate how these methods are being used to predict and manipulate cell fates, as well as their extension to various organisms (Fig. 5A).

### 6.1 Predicting key factors in disease-associated processes

One of the key reasons human diseases are often complex is the intricate interactions between immune cells (Schäfer *et al.* 2024), which cannot be fully captured by bulk methods. The advent of single-cell omics enables systematic analysis of immune cells, including the identification of distinct immune cell subsets (Wang *et al.* 2022b), the mapping of their developmental trajectories (Park *et al.* 2020), and the assessment of their responses to pathogens (Bossel Ben-Moshe *et al.* 2019), all of which hold promise for improving clinical predictions. For example, scRNA-seq has been used in bacterial infection models, such as Salmonella and tuberculosis (TB), to analyze immune responses (Bossel Ben-Moshe *et al.* 2019) (Fig. 5B). By deconvolving bulk measurements from mixtures of diseased cell types using scRNA-seq data from both healthy and infected samples, researchers have inferred disease induced, cell-type specific responses, revealing immune mechanisms linked to disease progression.

### 6.2 Cell state manipulation for cancer therapy and regenerative medicine

As scientists dissect the roles of plastic cell states/fates during development and tumor formation, the idea of manipulating cell states as a therapeutic strategy naturally emerges (Schade *et al.* 2024). Single-cell omics, especially in combination with genetic perturbation (Metzner *et al.* 2025) or lineage tracing (Schaff *et al.* 2024), are able to identify key factors as potential therapeutic targets (Fig. 5C). For instance, several epigenetic inhibitors are currently under development as part of cancer therapies (Comet *et al.* 2016). However, analysis of the single-cell RNA-seq map of breast cancer cells with specific epigenetic knockouts (KO) revealed that perturbation of H3K27me3 might trigger a partial epithelial-to-mesenchymal (EMT) transition, making cancer cells more migrative and aggressive (Zhang *et al.* 2022). Meanwhile, lineage-tracing-based research on melanoma has demonstrated that modulating signaling pathways prior to targeted therapy can reduce the number of drug-resistant cells (Harmange *et al.* 2023). Such a framework could also be applied to the study of somatic cell pluripotency, potentially improving the efficiency of reprogramming process (Jain *et al.* 2024).

### 6.3 Expansion of single-cell techniques to diverse organisms

Most of the single-cell omics techniques discussed earlier were initially demonstrated in mammalian cell lines and later applied to translational medicine research. However, the applications of single-cell techniques are not confined to specific organism. After making necessary protocol adjustments, researchers can extend these experimental strategies and computational algorithms to a variety of cellular systems (Alfieri *et al.* 2022). One translational research example in a non-model organism is in Gossypium hirsutum (Zhu *et al.* 2023), where lineage inference, gene co-expression network, and differential expression analysis were used to construct models of cell differentiation and gene regulation. The study identified LAX2, LAX1, and LOX3 as key genes involved in callus formation, which could serve as potential targets for enhancing the regenerative capacity of cotton cells (Fig. 5D). Beyond technical adaptations, expanding single-cell approaches to non-model organisms raises important biological questions about the conservation and divergence of cellular identity and fate programs across evolutionarily distant taxa (Sebé-Pedrós *et al.* 2018). Addressing these questions introduces associated computational challenges, such as establishing accurate gene annotation and functional equivalence across species without well-annotated reference dataset, and developing methods for cross-species data integration (Zhong *et al.* 2025). For instance, researchers recently constructed a unified single-cell atlas spanning six vascular plant species using an automated cell-type annotation tool named XSpeciesSpanner, revealing both evolutionarily conserved cell types (e.g. epidermal and vascular cells) and lineage-specific innovations (Xue *et al.* 2025).

## 7 Challenges and future directions

### 7.1 Modeling biological and technical variability

Biological systems inherently have diverse types of noise. In the context of single-cell gene expression, this noise can be broadly categorized as intrinsic or extrinsic (Elowitz *et al.* 2002, Swain *et al.* 2002, Sun and Zhang 2020). Intrinsic noise arises from the stochastic nature of molecular processes within individual cells (e.g. random fluctuations in transcription), and extrinsic noise stems from cellular heterogeneity that affect multiple genes simultaneously (e.g. variations in the abundance of shared transcription factors). However, distinguishing these two noise types remains challenging. Moreover, the timescales of the noise, or fluctuations, may vary in different genes, especially in mammalian cells (Huang 2009). Some genes display heritable variability that persists for multiple generations, potentially reflecting metastable cell states within the epigenetic landscape (Chang *et al.* 2008, Shaffer *et al.* 2020). This highlights the need for a comprehensive framework capable of systematically modeling the sources and timescales of biological variability including transient noise, heritable fluctuations, and stable heterogeneity (i.e. distinct cell types).

In addition to biological noise, technical errors and biases in omics measurements can further confound analyses. For example, in scRNA-seq, only a subset of RNA molecules is recovered and quantified, leading to additional Poisson-type technical noise or “dropout” events (Kharchenko *et al.* 2014). While several imputation methods have been proposed, accurately estimating the magnitude of true technical noise in the presence of biological variability remains difficult (Akhtyamov *et al.* 2023, Cheng *et al.* 2023). Furthermore, in lineage tracing or perturbation experiments, maintaining cell viability and functionality during genetic manipulation can affect the reliability of the results (Ecker *et al.* 2018). Consequently, ongoing improvements in single-cell techniques in reducing or quantifying technical variability are essential.

### 7.2 Data integration and interpretation

The advent of single-cell techniques has produced data for comprehensive characterization of cell states, but it brings in several challenges for data integration and interpretation. First, large-scale single-cell and multi-omics projects generate datasets of unprecedented size and heterogeneity, creating practical bottlenecks in storage, memory, I/O, and compute that influence algorithm design (Nouri *et al.* 2023, Zhao *et al.* 2024). Methods must be both computationally scalable (e.g. streaming and sparse representations) and transparent about which sources of variation they remove or retain, since it can affect dimensionality reduction and clustering (Stuart and Satija 2019).

The second challenge is cross-modality integration. In practice, most multi-omics dataset resources are “unpaired” (different assays measured in disjoint cell sets), and the information of cell identities will be inherently lost (Stuart *et al.* 2019). Scientists have attempted to project cells into co-embedded space to address this problem (Ghazanfar *et al.* 2024, Hao *et al.* 2024b), which requires reliable embedding algorithms and ground truth paired datasets for training. Conversely, “paired” assays can suffer reduced per-modality quality and coverage relative to single-omics experiments (Stuart *et al.* 2019, Zhang and Nie 2021). Each data type can also bring modality-specific technical and biological variabilities that complicate joint analysis (Rautenstrauch *et al.* 2022).

Lastly, even when integrating the same modality, biological interpretation is complicated by batch effects (i.e. from different samples, labs, platforms), and benchmark studies have shown a trade-off between removing batch effects and preserving true biological variation (Korsunsky *et al.* 2019, Tran *et al.* 2020, Luecken *et al.* 2022).

Large atlasing efforts demonstrate there is no one-size-fits-all integration strategy and that choices made during atlas construction strongly influence downstream interpretation (Hrovatin *et al.* 2025). To address these issues, efforts should combine careful experimental design and metadata standards with integration pipelines that (i) explicitly model sample/technology/lab effects, (ii) include metrics that quantify retention of known biological signals as well as batch removal, and (iii) expose parameters so users can tune the balance between harmonization and signal preservation. Building such context-aware, benchmarked frameworks will be essential for integrative analyses that remove technical artifacts while preserving biologically meaningful variation (Flores *et al.* 2023).

### 7.3 Toward an integrated experimental-computational paradigm

The convergence of experimental and computational approaches marks a paradigm shift in cell fate research. This integration creates iterative cycles where computational predictions guide experiments, and experimental findings drive new analytical methods, accelerating discovery in unprecedented ways.

The traditional linear workflow from hypothesis to experiment to analysis is being replaced by a dynamic cycle where computation and experimentation are deeply intertwined. Large-scale single-cell data now generate testable hypotheses, revealing unexpected cell states, novel regulatory circuits, and hidden transition pathways. These insights guide targeted perturbations, producing new data that drive innovative analyses. This bidirectional loop accelerates discovery: computational predictions highlight critical decision points for experimental validation, while unexpected experimental observations inspire new computational models, enabling rapid hypothesis testing and refinement.

Artificial intelligence, particularly foundation models, is revolutionizing our capacity to predict and control cell fate. AIVCs—comprehensive computational representations of cellular behavior—exemplify this integration of experimental and computational approaches. By enabling in silico perturbation experiments, AIVCs allow researchers to systematically explore thousands of genetic or chemical interventions, accelerating the identification of optimal reprogramming protocols and guiding cells toward desired fates. These models will also offer digital twin capabilities for drug screening, predicting off-target effects, and optimizing dosing strategies, supporting personalized medicine by simulating patient-specific cellular responses.

As experimental and computational boundaries blur, the most significant breakthroughs will emerge from fully integrated research programs. This unified approach promises to unlock therapeutic opportunities in developmental biology, regenerative medicine, cancer treatment, and beyond, fundamentally changing how we manipulate cellular identity for human health.
